# Global Dynamics in Protein Disorder during Maize Seed Development

**DOI:** 10.3390/genes10070502

**Published:** 2019-06-30

**Authors:** Jesús Alejandro Zamora-Briseño, Alejandro Pereira-Santana, Sandi Julissa Reyes-Hernández, Enrique Castaño, Luis Carlos Rodríguez-Zapata

**Affiliations:** 1 Unidad de Biotecnología, Centro de Investigación Científica de Yucatán, Calle 43, Número 130, Chuburná de Hidalgo, Mérida CP 97205, Yucatán, Mexico; 2 Centro de Investigación y Asistencia en Tecnología y Diseño del estado de Jalisco. División de Biotecnología Industrial. Camino Arenero 1227, El Bajío, Zapopan CP 45019, Jalisco, Mexico; 3 Unidad de Bioquímica y Biología Molecular de Plantas, Centro de Investigación Científica de Yucatán, Calle 43, Número 130, Chuburná de Hidalgo, Mérida CP 97205, Yucatán, Mexico

**Keywords:** Intrinsically disordered proteins, dehydration, seed development, maize

## Abstract

Intrinsic protein disorder is a physicochemical attribute of some proteins lacking tridimensional structure and is collectively known as intrinsically disordered proteins (IDPs). Interestingly, several IDPs have been associated with protective functions in plants and with their response to external stimuli. To correlate the modulation of the IDPs content with the developmental progression in seed, we describe the expression of transcripts according to the disorder content of the proteins that they codify during seed development, from the early embryogenesis to the beginning of the desiccation tolerance acquisition stage. We found that the total expression profile of transcripts encoding for structured proteins is highly increased during middle phase. However, the relative content of protein disorder is increased as seed development progresses. We identified several intrinsically disordered transcription factors that seem to play important roles throughout seed development. On the other hand, we detected a gene cluster encoding for IDPs at the end of the late phase, which coincides with the beginning of the acquisition of desiccation tolerance. In conclusion, the expression pattern of IDPs is highly dependent on the developmental stage, and there is a general reduction in the expression of transcripts encoding for structured proteins as seed development progresses. We proposed maize seeds as a model to study the regulation of protein disorder in plant development and its involvement in the acquisition of desiccation tolerance in plants.

## 1. Introduction

Over the last few years, new information has been generated regarding the role of intrinsically disordered proteins (IDPs) which has changed our understanding of protein biochemistry. These findings led to the categorization of IDPs as an interesting group of proteins which differ from globular proteins in terms of action modes and physicochemical characteristics. IDPs possess low complexity and a biased composition of amino acid (aa), being almost depleted in hydrophobic and aromatic residues while enriched in polar and charged aa [1]. These features in the primary structure confer them with a high net charge and low mean hydrophobicity [2]. For these reasons, such protein sequences are unable to fold into stable, rigid, globular, three-dimensional structures. In contrast, they are dynamically interconvertible ensembles of spatial conformations ranging from extended statistical coils to collapsed globules [1,3]. Some proteins are predicted to be entirely disordered (IDPs in *sensu stricto*), while others are not intrinsically disordered throughout, but have disordered segments (intrinsically disordered regions IDRs) [4]. The collection of IDPs/IDRs of a given organism in a specific condition can be collectively referred as disordome [5].

IDPs are enriched in signaling and regulation of key cellular processes, such as control of cell division, apoptosis, post-translational modification, transcription, etc. Bioinformatics analysis of the Swiss-Prot database reveals that at least 238 function keywords of cellular process is given between IDPs/IDRs [6,7,8]. Flexible conformation of intrinsic disorder confers advantages to proteins, such as an increased speed of interaction, the combination of specificity with weak and reversible binding, and the ability to carry out more than one function [9]. In this context, several advantages have been proposed for IDPs/IDRs, which include, economizing genome and protein resources, overcoming steric restrictions in binding, achieving high specificity with low affinity, facilitating post-translational modifications, enabling flexible linkers, preventing aggregation, providing resistance to non-native conditions and finally, allowing compatibility with more available sequences [6].

In plants the genomic-wide information available regarding IDPs is rather limited in comparison to other eukaryotic organisms and it is circumscribed to *Arabidopsis thaliana* [10,11], and a few other plant models [12,13,14]. The fact that many IDPs can serve as signal integrators in signaling cascades and stress-response processes, allows the supposition that their role can be especially important in plant development and adaptation to environmental conditions, probably by providing them with a fast mechanism to obtain complex and highly interconnected molecular networks [15]. It has been pointed out that several IDPs in plants play key roles in response to different stresses [10,11], in particular water stress [16,17,18]. It has been suggested that plants may use IDPs independently to adapt quickly and efficiently to environmental changes from which they cannot escape [10].

Although important progress has been achieved in our comprehension of the functions of disordered proteins in plants, the answers to many interesting questions remain elusive. An important feature to be evaluated is their role in desiccation tolerance (DT) in plants. When cellular water content is reduced, the density of macromolecules is increased, an effect known as macromolecular crowding [18]. In some cases, this density must be significantly adjusted, for example, in orthodox seeds in dry state. 

The end of orthodox seed development is typified by a programmed period of dehydration leading to the loss of bulk water from the entire structure [19]. Such dehydration causes a reduction in the cellular volume. The macromolecular crowding resulting from the cytoplasmic compaction, provide an environment amenable to numerous undesirable interactions that can lead to organelle-cell membrane fusion, and denaturation and aggregation of certain proteins especially susceptible to such conditions [20]. Acquisition of desiccation tolerance, or the ability to withstand these very low water potentials and consequent macromolecular crowding, has been correlated with the accumulation of various protective compounds including protective proteins and sugars [19]. Additionally, it has been proposed that a possible mechanism to partially help cope with this effect is the induction of proteins less prone to the loss of function induced by denaturalization [5]. Furthermore, the induction of IDPs could represent a compensatory mechanism to mitigate the effect of the macromolecular crowding in the cell, since these proteins are not able to lose their function by a denaturalization-mediated mechanism. In addition, a reduction in the content of structured proteins with propensity to denaturalization, mediated by the lack of water, is also feasible. 

Understanding the spatio-temporal expression pattern of genes represents an invaluable source of information regardless if a gene contributes or not to its function in the cellular context [21,22]. Therefore, the expression profile of coding transcripts under a desiccation context can shed light on the relationship of protein intrinsic disorder with such characteristics. In these sense, orthodox seeds may be particularly useful models to evaluate association between the overall regulations of protein disorder under a dehydration context, since these are specialized structures which have developed evolutionary conserved adaptations to resist the extreme loss of water [23]. A very well study orthodox seed is the maize caryopsis. This is an amenable plant model, with a fully sequenced and well annotated genome [24], with several available public transcriptomic datasets of seed development, as well as a detailed description of the anatomical, physiological and biochemical changes occurring during this period [25,26,27,28].

For these reasons, in this work we tested the hypothesis that a desiccation context favors the accumulation of proteins less prone to denaturation mediated by water loss. In this sense, we considered that the increase of protein disorder could represent a strategy to increase the general resilience of the proteome as a response to extreme water loss conditions. To evaluate this idea, we analyze the expression of genes according to the disorder content of their coded proteins during maize seed development. We found that the overall disorder content differs throughout the process of seed development and it is closely associated with the different developmental stages in which maize seed is divided. Interestingly, a general reduction in the content of genes encoding for structured proteins can be observed. We identified several disordered transcription factors (TF) involved in key regulatory activities during seed development. We also identified clusters of highly disordered proteins, the expression of which is correlated with the onset of the program which leads to the acquisition of dehydration tolerance in seed. Therefore, we consider maize seed as a good model to study the IDPs roles and understand their implications during development as well as in drought stress response in general. Finally, we proposed a general model that involve an overall reduction of the structured proteins and an increase in the content of IDPs as a mechanism to reduce the negative impact of extreme water loss in the cell. 

## 2. Materials and Methods

### 2.1. Transcriptomic Data Acquisition

To correlate if there is a dependency between the disorder content of the proteins codified by transcripts during seed development progression, we used the available transcriptomic data of whole maize seed during its development. Transcriptome data was downloaded from the EMBL-EBI Expression Atlas (https://www.ebi.ac.uk/gxa/home). This data was generated by Chen et al. [25] and corresponds to the experiment identified as “Transcription profiling by high throughput sequencing of maize embryo and endosperm during development”. This data includes the counts of transcripts standardized in Transcripts per million readings (TPM) of maize seed from day 0 until day 38 of post-pollination (DAP). 

The identifiers of each transcript were retrieved from the expression table to obtain the protein sequences codified by each transcript. The maize genome B73 version 4 was used in this study, and the file annotations were downloaded from Ensembl Plants (plants.ensembl.org/index.html). 

### 2.2. Protein Disorder Analysis and Data Clustering 

The prediction of the protein intrinsic disorder was performed using Espritz [29] setting X-ray and Best Sw as parameters. The longest isoforms of each transcript were used and cataloged according to the percentage of structural disorder of their encoded protein. Thus, transcripts were cataloged into four different categories from lower to higher percentage of structural disorder (their encoded proteins) as follows: (I) 0–25, (II) 25–50, (III) 50–75, and (IV) 75–100 of disorder percentage. The overall protein sequences of each category were analyzed to quantify the relative composition of aa. The percentage of aa composition of each category were plotted as a heatmap. This data was also used to perform Principal Component Analysis (PCA) to identify if the categories could be distinguished from each other. Both analyses were performed with ClustVis [30]. Proteins were annotated with Blast2GO [31] and gene ontology (GO) enrichment analysis was performed with WEGO [32]. Enriched GO terms were exported and plotted as a heatmap with ClustVis [30]. To correlate protein length and structural disorder, we classified the proteins into six categories according to their length: (I) 30–250, (II) 250–500, (III) 500–750, (IV) 750–1000, (V) 1000–1500, and (VI) > 1500 aa. Subsequently, we classified our four protein disordered categories according to their length.

### 2.3. Transcriptome Mining and Differential Expression Analysis

To obtain the proportion of transcripts which encode for disordered proteins with respect to the structured proteins, we obtained the relative content of transcripts of each category. The total sum of TPM of each category was obtained and divided by the total number of TPM per day and multiplied by 100, and the results were plotted with respect to the time (0–38 DAP). These kinetics were divided into five different developmental stages (R1, R2, R3, R4, and R5), according to Hanway [33]. Additionally, the number of expressed genes of each category (considering as expressed genes those with a TPM > 0) was quantified and represented as percentage. To confirm the correlation between the developmental stage and the global expression profile of IDPs, a Pearson’s correlation analysis of transcripts encoding for proteins with a disorder content > 25% was depicted.

Venn diagrams of the expressed transcripts (TPM > 0) were constructed to identify intersected and specific transcripts between developmental stages. To simplify the clustering of the data, the transcripts were divided into transcripts encoding for structured proteins (0–25% of disorder) and transcripts encoding for IDPs (>25% of disorder). To obtain transcripts that encode for highly disordered proteins (>75% of disorder) which are also differentially expressed, the EdgeR package was used [34,35], using a cut-off value of false discovery rate (FDR) of 0.001. From these data, we defined two groups of transcripts which encode for IDPs, the transcripts that are positively regulated and those that are negatively regulated throughout the development of the seed. Additionally, a functional enrichment analysis was carried out, for both the up- and the down-regulated genes identified. This analysis was performed with Blast2GO [31]. 

### 2.4. Gene Co-Expression Network Analysis

To find out if functional specialization of TFs exists based on their level of disorder, we inferred the regulatory networks formed according to structural disorder of TFs identified as up-regulated in early (R1), middle (R2 and R3) and late (R4 and R5) stages. Up-regulated transcription factors were obtained from the differential analysis with EdgeR, and TF expressed in seed identified previously [25]. 

Transcript per million reading values of genes corresponding to seed dataset were filtered to reduce complexity and data noise. For the co-expression matrix training, we just used those genes with at least one count per time-point treatment and with a total sum of counts per treatment greater than 21 (there were 21 time-point treatments). To build the pairwise co-expression matrix, we use the GENIE3 Bioconductor package [36,37], which is based on the Random Forest machine-learning model [38] with default parameters. We build four different co-expression networks using as “Regulator nodes” the 73 differential expressed transcription factors (DETF) found in our differential analysis and the filtered seed TPM matrix as “Target nodes”. The 73 DETF were grouped according to their disorder level (0–25, 25–50, 50–75, and 75–100% of intrinsic disorder). For the first network, we used as regulator nodes the five DETF with 0–25% of disorder; for the second network we used 34 DEFT within a 25–50% of intrinsic disorder; the third network was built on 22 DETF with 50–75% of intrinsic disorder; and the fourth network was built on 12 DETF within a 75–100% of intrinsic disorder. The resulting files from GENIE3 software were loaded as tables into Cytoscape v.3.6.0 [39]. All the co-expression networks were taken as undirected but weighted. The networks were clustered by using the GLay clustering algorithm plug-in in Cytoscape [40] according to topological edge connections. Finally, transcripts clusters were separated according to TF disorder level and stage of development in which their expression is induced. 

## 3. Results 

**Figure 1:**
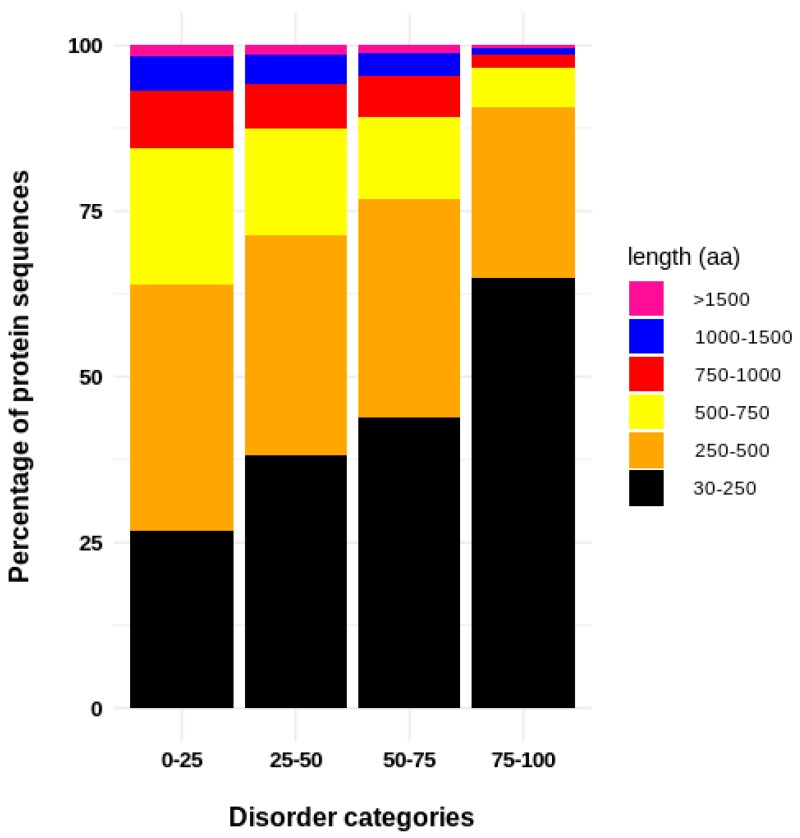
Relationship between the level of disorder and the protein length. It can be observed that the more disordered the proteins, the greater the relative proportion of smaller sized proteins constituting each category.

**Figure 2:**
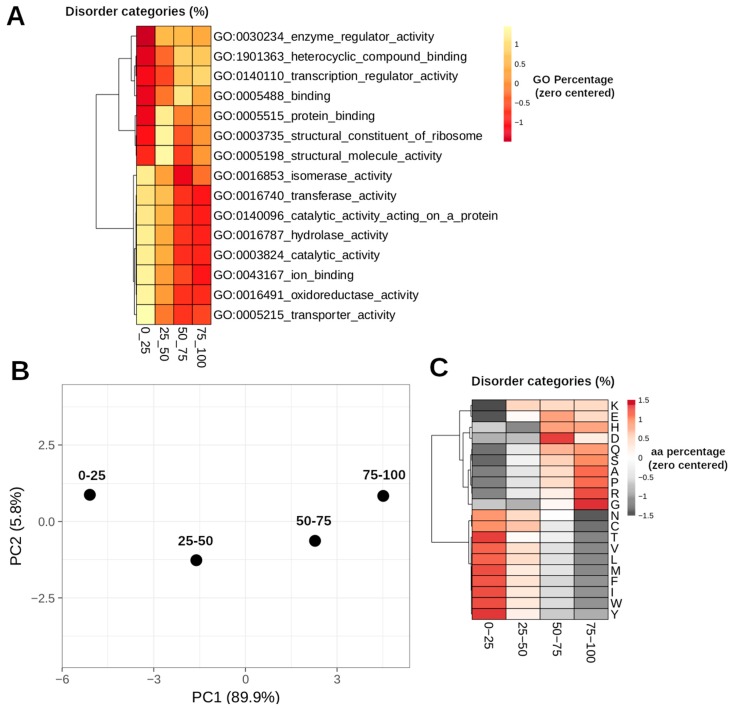
General characteristics of the proteins according to the disorder content of each category defined in this work. (**A**) Heatmap of the enriched Gene Ontology (GO) terms between the four defined categories for a *p*-value < 0.01. Colors represent the percentage of each GO category with scaling applied to rows and the values centered to zero. (**B**) Principal Component Analysis (PCA) analysis of the aa composition of the proteins that constitute each category. Unit variance scaling is applied to relative aa composition of the proteins of each category; Singular Value Decomposition (SVD) with imputation is used to calculate principal components. X and Y axis show principal component 1 and principal component 2 which explain 89.9% and 5.8% of the total variance, respectively. (**C**) Heatmap of the aa composition of the different categories of protein disorder. The rows were centered, and scaling was applied. The rows were then clustered using correlation distance and complete linkage.

**Figure 3:**
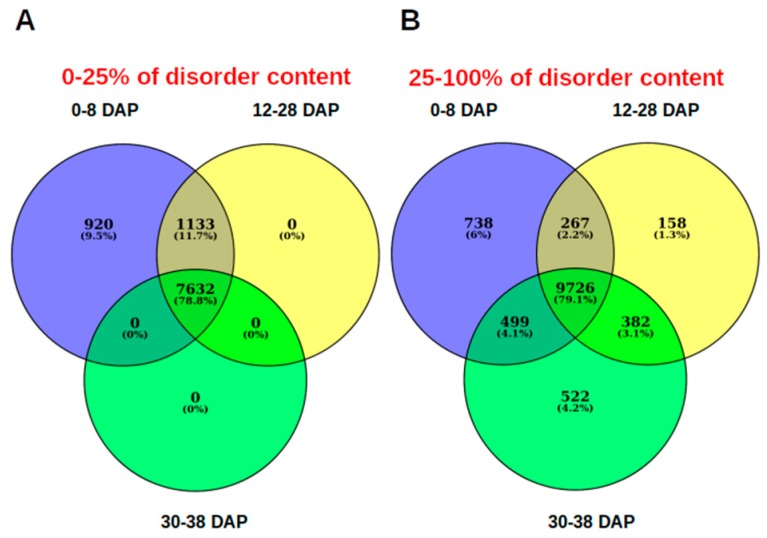
Venn diagrams of the shared expressed genes (TPM -Transcripts Per Kilobase Million- > 0) of each stage, according to the disordered content of the proteins codified by each transcript. (**A**) Transcripts encoding for structured proteins (0–25% of disorder content) is expressed in the early (0–8 Days After Pollination -DAP-) and then are turned off in the following stages. (**B**) There are transcripts encoding for intrinsically disordered proteins (IDPs) which are expressed specifically in each stage.

**Figure 4:**
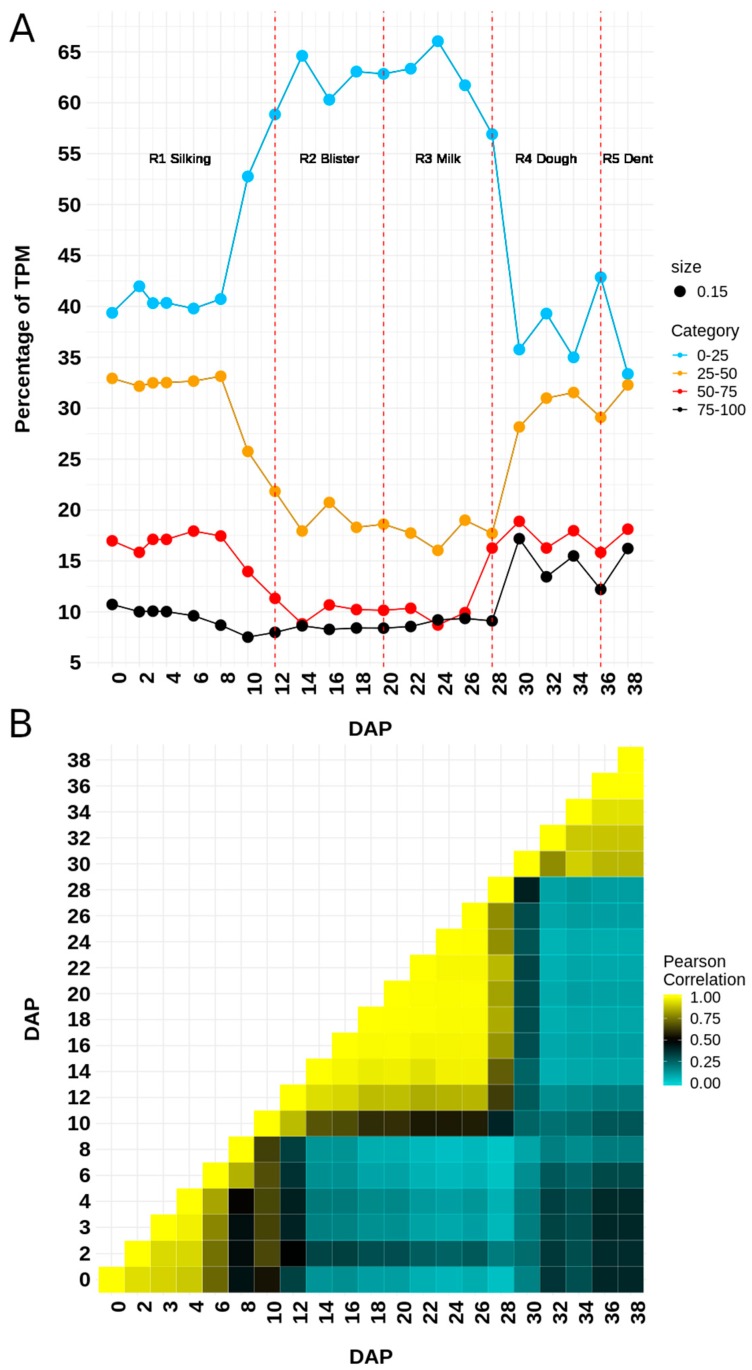
Expression patterns of transcripts according to the disorder content of the protein that they encode. (**A**) Percentage of TPM of transcripts encoding for proteins with different degrees of protein disorder. There is an induction in the overall expression of transcripts encoding for structured proteins during the middle phase of seed development. (**B**) Pearson’s correlation analysis of the expression of transcripts encoding for proteins with > 25% of disorder. There is a clear separation of the different phases of development, which suggests a marked specific expression of transcripts during each of the developmental stages.

For the analyzed dataset, we found a clear relationship in the protein length and the protein disorder content (Figure 1). The proportion of proteins with smaller length is greater when the disorder increases. Thus, the IDPs tend to be shorter in comparison with the structured proteins. From a functional point of view, there is a clear separation between the enriched functions of structured (=<25% of disorder) and disordered proteins. Disordered proteins are enriched in regulatory function, in contrast to structured proteins which are enriched in catalytic functions (Figure 2A). From a structural point of view, it is possible to separate four categories based on aa composition, since we can differentiate structured proteins (< 25% of protein disorder) from moderately disordered (25–50% and 50–75% categories) and highly disordered proteins (>75% of protein disorder; Figure 2B). This separation is clearly explained given that structured proteins tend to be enriched in hydrophobic residues, while polar and hydrophilic aa are over-represented in highly disordered proteins (Figure 2C). However, during the progression of seed development, there is a reduction in the number of expressed genes (TPM > 0) encoding for structured proteins (Figure 3). Apparently, the genes that encode for structured proteins are activated in the first stages of seed development and they are inhibited later in the course of development (Figure 3A). In contrast, this does not occur for transcripts that encode for proteins with >25% structural disorder. In this case, there is a subset of genes that are specifically activated in each of the developmental stages (Figure 3B). According to our analysis, the relative content of transcripts encoding for structured proteins (=<25% of disorder) and IDPs is not constant throughout seed development. If we divide the dataset into the five different phases of seed development (R1, R2, R3, R4, and R5), which include the period of time analyzed in this work (0–38 DAP), it is highly interesting to note that in the R2 and R3 phases, there is an important increase in the proportion of transcript counts of structured proteins (=<25% of protein disorder; Figure 4A). Therefore, the most abundant transcripts of these phases were identified, and the 15 most abundant ones were selected. Thus, the following genes were identified: alpha-zein A20 (Zm00001d019155), alpha-zein 19D1 (Zm00001d030855), zein 15kD (Zm00001d035760), zein 10 kD (Zm00001d045937), alpha-zein3 22kD (Zm00001d048809), alpha-zein4 22kD (Zm00001d048812), alpha-zein (Zm00001d048813), zein 3 (Zm00001d048817), alpha-zein 19B1 (Zm00001d048848), alpha-zein (Zm00001d048849), alpha-zein PMS1 (Zm00001d048850), alpha-zein z1C2 (Zm00001d049243), and alpha-zein Z1A (Zm00001d049476). Total counts of these highly expressed transcripts represent up to 39.3 ± 7.94 (mean ± SD) percent of total TPMs in middle phase (R2 and R3), while in early and late phases their counts represent 0.62 ± 0.84 and 2.70 ± 3.43, respectively. 

Moreover, the expression of IDPs (>25% of protein disorder) is highly dependent on the developmental phase, and the global expression of this type of transcripts is correlated with developmental time (Figure 4B). The complete list of expressed transcripts classified in the different categories of disorder, as well as their expression values are shown in Supplementary Tables S1–S4.

It is evident that transcriptomes from 0 to 10 DAP (R1), 12 to 28 DAP (R2 and R3), and 30 to 38 DAP (R4 and R5) formed clusters, which correspond to early, middle, and late phases of development, respectively [25]. R1 is an active period of cell division and cell elongation, while R2 and R3 correspond to morphogenesis and maturation phases of development, respectively. During the R2 and R3 stage, the embryo undergoes active DNA synthesis, cell division, and differentiation, followed by synthesis of storage reserve and desiccation [41]. The distinct cluster after R4 and R5 stages (30 to 38 DAP) encompass the end of storage compound accumulation in the endosperm and the activation of biological processes involved in dormancy and dehydration [25].

**Figure 5:**
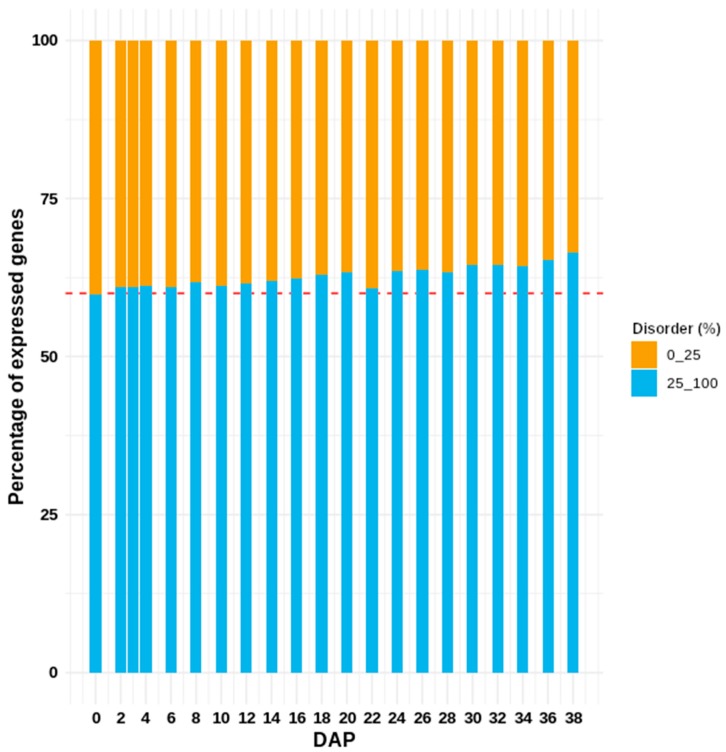
Dynamics in the relative composition of transcripts expressed throughout seed development. It is a clear reduction in the relative composition of structured proteins. The atypical data observed in the 22 DAP coincides with the peak of seed filling.

In fact, at the end of the R5 stage the proportion of expressed transcripts encoding IDPs is increased with respect to the transcripts encoding for structured proteins (Figure 5) with an atypical peak of transcripts encoding for structured proteins at 22 DAP. Thus, it is expected that the mean length of the proteins in the late phase of seed development tends to become shorter in comparison with the beginning of the embryogenesis. 

**Figure 6:**
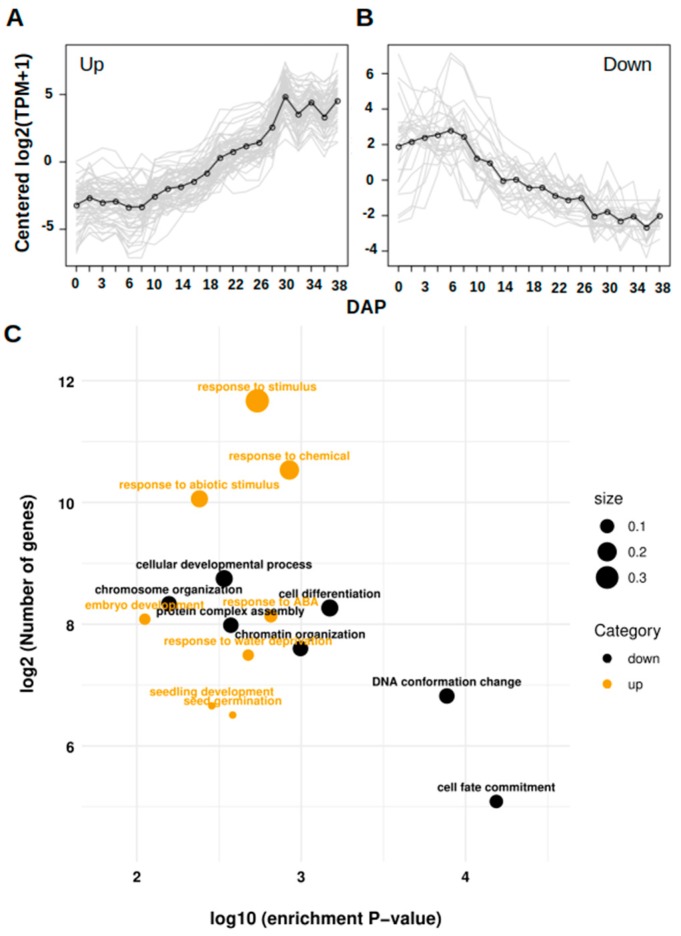
Gene expression profile of highly disordered IDPs with more than 75% of disorder in its sequence. (**A**) and (**B**) There is a group of IDPs which are induced and another group which are actively repressed throughout the period evaluated. (**C**) Functional enrichment analysis for each up and down-regulated gene encoding for highly IDPs. The size is proportional to the number of enriched genes among the total number of their GO term associated genes.

These results suggest that the changes in the proportion of structural disorder are a consequence of the functional changes occurring at the different developmental phases. When seed development begins, a large quantity of cellular resources is advocated to cell division and cell differentiation. This process requires an important gene regulation. However, when the seed enters the middle phase of development, the metabolism becomes primarily biosynthetic, given that several enzymes are needed for the synthesis of starch and other storage compounds, and storage proteins synthesis take place. At the end of the filling phase and the beginning of the late stage of development (R5), the onset of the dehydration program takes place. For this reason, we were advised to identify the up-regulated transcripts that codify for IDPs. We identified 1015 transcripts codifying for IDPs (>25% of disorder) which are positively expressed at the end of the R5 stage. Between these IDPs-coding transcripts, we identified 193 up-regulated transcripts that codify for highly disordered proteins (> 75% of protein disorder). In contrast, 157 transcripts were identified as down-regulated transcripts which codify for this type of proteins, but are highly expressed during early embryogenesis (Figure 6A,B). Interestingly, the GO enrichment analysis of both sets of proteins reflects the switch in the metabolism occurring during maize seed development (Figure 6C). The gene ontologies associated with the up-regulated genes are clearly different from those associated with the down-regulated genes. In the down-regulated group, several GO terms associated with cell development and cell differentiation are enriched. Among these GO terms, some are representative, such as “Cellular developmental process”, “Chromosome organization”, “Cell differentiation”, and “DNA conformation change”. The up-regulated transcript set shows enrichment of GO terms associated with plant adaptation to drought conditions which become obvious. Some over-represented processes are “response to abiotic stimulus”, “Response to water deprivation”, “Response to ABA”, “Seed germination”, and “Embryo development”. 

In the group of down-regulated genes, there were identified genes encoding disordered proteins involved in DNA regulation and RNA regulation such as Histone H1 (Zm00001d013066 and Zm00001d013067), and Histone H1a (Zm00001d018981 and Zm00001d034479), Histone H2A (Zm00001d044246), High mobility group protein 3 (Zm00001d051427), High mobility group family A (Zm00001d032239), nucleosome/chromatin assembly factor D (Zm00001d052749), H/ACA ribonucleoprotein complex subunit 3-like protein (Zm00001d051116), H/ACA ribonucleoprotein complex subunit 1-like protein 1 (Zm00001d052952). Interestingly, there were identified several transcripts participating in cell signaling and TFs involved in development such as Calmodulin binding protein (Zm00001d002630 and Zm00001d028841), Calreticulin-2 (Zm00001d005460), Calmodulin binding protein isoform 2 (Zm00001d038838), Protodermal factor 1 (Zm00001d043588), Early nodulin 75 protein (Zm00001d031878), HMG-transcription factor 13 (Zm00001d021433), Homeobox-transcription factor 41 (Zm00001d017422), and WRKY-transcription factor 48 (Zm00001d015515). Meanwhile in the list of up-regulated genes encoding for highly disordered proteins, we identified several TF involved in stress response and dormancy, including bHLH-167 (Zm00001d003677), MYB-139 (Zm00001d005300), bZIP-91 (Zm00001d007042), MYB-related-111 (Zm00001d026017), DRE-binding protein 1 (Zm00001d032295), bZIP-29 (Zm00001d034571), bHLH87 (Zm00001d038863), ZF-HD-9 (Zm00001d044662), bZIP (Zm00001d052562), HSF-11 (Zm00001d034433), as well as several proteins involved in seed maturation, protection of cellular components, and water stress response, such as Dormancy-associated protein homolog 3 (Zm00001d047503), LEA 3 (Zm00001d043709 and Zm00001d038870), Xero 1 (Zm00001d043730), Seed maturation proteins (Zm00001d044022, Zm00001d024414, Zm00001d033782, and Zm00001d035000), and glycine-rich cell wall structural protein (Zm00001d017033). The complete list of up-regulated and down-regulated genes encoding for highly disordered proteins (> 75% of protein disorder) from day 0 to day 38 post-pollination are presented in Supplementary Table S5; Table S6.

**Figure 7:**
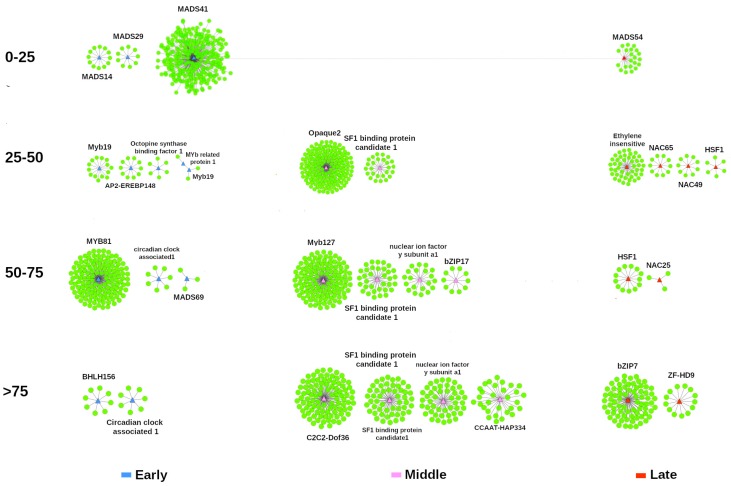
Co-expression networks analysis using up-regulated TF as regulators. Differentially expressed TFs of each phase were used to infer the key TFs, according to their disorder content. TFs are preponderantly IDPs. At early stage, TFs families are associated with developmental process. During the middle phase, key TFs are advocated to control of biosynthetic processes, such as accumulation of storage proteins. At the beginning of late phase, TFs families are preponderantly focused on the control of desiccation acquisition mechanisms. Only TFs with more than two target genes were included in the figure.

Using the co-expression networks analysis, an important number of key transcription factors were identified forming regulatory clusters of transcripts. As expected, most of the TFs identified forming clusters have a certain level of structural disorder (Figure 7). Although during the middle phase there is an increase in the total content of transcripts that encode for structured proteins, in this period we did not identify any structured TF forming regulatory clusters. Likewise, structural disorder seems to play a preponderant role in some TF families, including AP2, NAC, bZIP, HSF, Opaque 2, and Myb families.

Interestingly, in this analysis we identified several TFs of NAC, ethylene-insensitive, HSF, and bZIP families as key regulators of the late phase. The general list of over-regulated TF that form regulatory clusters, as well as their target genes, is presented in the Supplementary Table S7.

In general, the data generated in this work suggest that there is an active adjustment in the content of the disorder content during the maize seed development.

## 4. Discussion

A potent feature of the transcriptomic studies is the ability to derive conclusions of the mechanisms that are being regulated under a defined biological condition. A complementary approach would be to analyze the gene expression in relation to predicted conformational properties of the proteins they encode. However, the use of such approach is not so exploited [42]. This kind of information is not obvious and must be quantified. The consideration of the global structural characteristics of the proteins that are synthesized under a given condition could give us additional information regarding the adaptive mechanisms used by organisms to respond to their environment. For example, recently it was corroborated that proteins, whose expression levels increase by heat in *A. thaliana*, are enriched in charged aa and have a low proportion of polar and hydrophobic aa, in comparison with the proteins that are repressed^11^. The regulation of the types of proteins based on their aa composition could represent a kind of adaptive response to environmental changes which have not been previously taken into account.

There are enough examples of the accumulation of IDPs in response to loss of water, to suggest that the increase in structural disorder is part of the responses to water stress [20,43,44,45,46,47]. For example, in orthodox seeds, LEA proteins (a group of very well characterized IDPs) are highly abundant during the late stages of plant seed development when the embryo becomes desiccation tolerant and its induction coincides with the onset of desiccation tolerance [44,48]. Previously, it was pointed out that there is a strong correlation between the desiccation and intrinsic disorder in proteins, since IDPs are involved in vitrification, water replacement, molecular shielding, membrane stabilization, preservation of cellular organization and structure, water retention, and scavenging of reactive oxygen species [46]. Another feasible measure that cells can implement to reduce the effects of lack of water, is the reduction of proteins which are more sensitive to loss of function mediated by lack of water. Based on these ideas, in this work we analyzed the regulation of the expression of transcripts according to the disorder content of the encoded proteins along the maize seed development, including the beginning of late phase.

During maize seed development there is a progression of a morphogenetic program which includes the acquisition of desiccation tolerance at the end of the developmental program. This is accompanied by a sharp reduction in the relative content of water. Interestingly, in this work we found that this is also accompanied by a reduction in the number of genes encoding for structured proteins. To our knowledge, this is the first global survey of the expression of transcripts according to the structural disorder of their encoded proteins under a context of seed development and water reduction.

We first described general features of the different categories in which proteins were divided according to their disorder content. It is interesting to note that there is a clear relationship between the protein disorder level and the protein length. To our knowledge such association has not been pointed out previously. However, it was found that the percentage of occurrence of most of the residue types depends significantly on protein dimension [49], but this dependency was not explained. The functional specialization of the IDPs with respect to the structured proteins may help to explain such observations. The GO enrichment analysis showed that the protein specialization is dependent on the level of protein disorder. IDPs are specialized in regulatory activities, while the structured proteins are enriched in catalytic functions. It has been observed that transcription factors possess a high degree of disorder in the form of intrinsically disordered regions (IDRs) [50]. In fact, disorder predictions show that 83–94% of all known TFs possess extended regions of disordered residues [51]. This explains why most of the TFs identified in the co-expression analyzes fall into some category of disorder. 

Disorder is less frequent in enzymes and many proteins involved in catalytic activity are structured [52]. This functional bias can be explained if we take into consideration that intrinsic disorder is a direct consequence of the particularity of the amino-acid composition of IDPs. Compositional bias is a common attribute of IDPs [2,8,53,54]. IDPs are generally rich in Arg, Gln, Glu, Lys, Pro, and Ser, while they are deficient in Cys, Ile, Leu, Phe, Trp, Tyr, and Val [4]. These data are in accordance with our predictions, which show a clear difference for each protein category defined in this work. 

We found that transcripts encoding for structured proteins are actively induced during the middle seed filling phase (R2 and R3 stage), and coincides with the biosynthesis and accumulation of reserves^25^. During this phase, the conversion of imported sucrose and aa into starch and storage proteins can account for about 90% of kernel total dry matter, with the most rapid grain-filling occurring between 21 and 25 DAP, regardless of the genetic background [26,55,56,57]. A phenomenon that characterizes this period is the accumulation of zeins in endosperm. Zeins are a class of storage proteins which have been classified as α-, β-, γ-, and δ-zeins based on its solubility and its structural properties [58,59,60,61]. The α-zein protein group is the largest among them, consisting of ∼ 80% of the total zeins proteins [62]. They are unusually rich in hydrophobic amino acids including leucine, alanine, and proline, alongside the hydrophilic glutamine [59]. In general, zeins are highly insoluble in water, and are structured proteins [60,63]. For example, 22kDa and 19kDa zeins are conformed of nine contiguous, topologically antiparallel helices grouped within a distorted cylinder [64]. In this work it was found that a large part of the increase in the content of transcripts that encode for structured proteins can be explained by the incredible increase in the expression of the genes of the zein family. The transcriptional control of genes encoding 22kD zein proteins is mediated by opaque-2 (o2), a well-known TF that is a key transcriptional regulator during the middle phase [65]. Interestingly, Opaque-2 (Zm00001d018971) was identified as a regulator of several zeins in our networks analysis which help to support our predictions. 

On the other hand, the number of expressed genes encoding for structured proteins shows a continuous reduction after middle phase. At the beginning of the maturation phase (R5 stage), there is a subset of highly disordered proteins which is actively induced. However, the regulation of these genes starts at the R4. The regulation of the desiccation program begins immediately after the end of the most active phase of biosynthesis, before the acquisition of tolerance to desiccation becomes a visible characteristic. This is a phase that can be considered to be preparatory. Prior to R5, kernels along the length of the ear begin to dent or dry on top. Moreover, in the R5 stage the kernels are drying and moisture content is severely reduced, reaching less than the 50% [5,33]. In this stage, a decreasing grain-filling rate culminates in physiological maturity and black-layer formation, and is related to seed dormancy [26]. In addition, our data suggest that there is a continuous reduction in the number of transcripts encoding for structured proteins. Interestingly, there is an atypical peak in the relative content of this kind of transcripts at 22 DAP, which coincides with the maximum starch accumulation rate, occurring at 21 DAP [26,57]. 

The early stage characteristically involves cell division, after which the endosperm cells enlarge and as a result of several metabolic processes acquire storage proteins and starch [66]. In the final phase of embryogenesis, desiccation tolerance is acquired, and dormancy is established [67]. Therefore, differential expression analysis is very useful to define the IDPs associated with the first developmental process and those associated with the beginning of the maturity phase. Within these two types of transcripts, the specialization of the IDPs can be differentiated, since their ontologies are involved in clearly different processes. While the down-regulated IDPs genes are dedicated to developmental-associated processes, the induced ones are involved in functions related to the abiotic stress and environment stimulus responses. 

Within the IDPs identified in the first category, it is interesting that there are several proteins associated with nucleic acid packing, such as histones and high mobility group (HMG) proteins. It is recognized that many protein functional classes are heavily dependent on intrinsic disorder. Among these disorder-centric functions are interactions with nucleic acids and protein complex assembly. Previously, it has been demonstrated that all the members of the histone family are IDPs. In fact, intrinsic disorder is necessary for various histone functions, starting from heterodimerization to formation of higher order oligomers, to interactions with DNA and other proteins, and to post-translational modifications [68]. HMG proteins are nuclear proteins that binds transiently to nucleosomes, changes the local architecture of the chromatin fiber, and affects several DNA-related activities such as transcription, replication, and DNA repair [69]. HMGs are highly disordered proteins, and they are mainly random coiled in solution, but they are subjected to folding upon binding with DNA or other interators [70,71]. 

In addition, we identified many down-regulated transcripts encoding proteins involved in plant development and calcium-mediated signaling, such as auxin-repressed protein (ARP) and Calreticulin. Evidence has emerged for ARP family members as IDPs. ARP genes are responsive to hormones involved in responses to biotic stress, such as salicylic acid (SA) and methyl jasmonate (MeJA), as well as to hormones that regulate plant growth and development, including auxins [72,73,74]. Calreticulin (CALR) is well recognized as a Ca^2+^-binding protein molecular chaperone that assists the folding of newly synthesized glycoproteins and modulates the Ca^2+^ homeostasis in the endoplasmic reticulum (ER) lumen [75]. In the ER, CALR binds not only Ca^2+^, but also interacts with many ER proteins, and with some mRNAs, thereby determining their fate. The potential functions of CALR are so numerous that identification of all of them is becoming a nightmare. Interestingly, its functional versatility is provided by its intrinsic disorder properties [76,77,78,79].

In contrast, up-regulated genes are over-represented by an important number of IDPs families, such as LEA proteins, dehydrins, glycine-rich proteins, proline rich proteins (PRPs) and Seed Maturation Proteins (SMP), which are implied in the DT and storability of vital seeds [80,81,82]. For example, LEA proteins, especially group 3 are highly hydrophilic, IDPs, whose expression are associated with the acquisition of desiccation tolerance in maturing seeds [20,81,83]. In maize, LEA3 gene was discovered to be responsive to ABA and hyperosmolarity and afterwards it was proved to be able to reduce cell shrinkage effects under dehydration [84]. On the other hand, PRPs are IDPs that were first identified as proteins that accumulate in the cell wall in response to physical damage. Members of the PRP gene family are expressed during leaf, stem, root, and seed coat development, seedling growth, and in cell types associated with lignification [85]. Functionally, PRPs are insolubilized in the cell walls with the involvement of reactive oxygen species (ROS)-mediated cross-linking and it play a role in the structural integrity of plant cells, participate in defense related activities, and plant cell surface interactions [85].

Glycine-rich proteins genes regulate diverse cellular processes in plant development and stress response. In tobacco, NtGRPs are highly regulated under osmotic stress and AtGRPs and OsGRPs have been identified acting as RNA chaperones that regulate mRNA export from the nucleus to the cytoplasm under stress condition. Also, GRPs proteins are important for the regulation of some steps in RNA post-transcriptional processing as splicing and polyadenylation [86,87,88]. The detection of these recognized IDPs, whose involvement in DT has been widely studied, can help us to support our disordered predictions. However, there are other IDPs that are induced in this phase, such as remorins and metallothioneins which have been associated with drought tolerance [89,90,91]; however, information regarding their relationship with DT is still limited, so that a more detailed analysis of its implications in the acquisition of tolerance to desiccation should be addressed. 

In our co-expression analysis, we detected several key TFs for each stage of development. Key TFs identified in the early stage are mainly specialized in coordinating processes related to cell development and cell differentiation, such as MADS and MYB TFs. MADS TFs are recognized to be involved in controlling many developmental processes in flowering plants, ranging from pollen and embryo sac development to root, flower, and fruit development [92]. Likewise, MYB proteins are key factors in regulatory networks controlling development, but also metabolism and responses to biotic and abiotic stresses [93]. 

In the middle phase, we identified several key intrinsically disordered TFs involved in the accumulation of storage reserves. Within this group of TFs, the identification of opaque2 is particularly important, due to its importance in the regulation of the accumulation of reserve proteins and its incredible capacity to integrate N and C metabolism [94]. Opaque 2 belongs to the basic leucine zipper (bZIP) class that is specifically expressed in the endosperm activating the expression of 22-kDa α-zein and 15-kDa β-zein genes. Opaque2 also directly or indirectly regulates several other non-storage protein genes, such lysine-ketoglutarate reductase and heat shock protein 70 [95,96]. In our network analysis, these and others important proteins, such as ABA receptor PYL5, NAC87, 60S ribosomal protein L7-1, 40S ribosomal protein S24, giberellin-2 oxidase 1, aconitase1, TFDII subunit 9, among others were predicted to be regulated by opaque2 TF.

During the late phase, most of the regulator identified belongs to NAC TF family. The NAC TFs comprise one of the largest family of TFs in plants [97,98]. The NAC TFs play a vital role in the complex signaling networks during plant stress responses. It appears that an important proportion of NAC genes function in stress response according to the expression data from global expression analyses in many plants [99,100,101]. Interestingly, Heat shock factor 1 (HSF1) (Zm00001d005888, 50–75% of disorder), appears to be involved in the regulation of oxidative stress, as many redox-related proteins were identified as regulated by this TF. For example, we identified proteins such as wound induced protein, respiratory burst oxidase, glutaredoxin subgroup III protein, glutaredoxin, and 17.4 kDa class I HSP. In tomatoes was found that HsfA1 is the master regulator in response to heat shock stress [102], and it has been proposed that some members of HSFs family may act as redox sensors [103].

On the other hand, TF bZIP7 seems to play an essential role in this stage. Moreover, to our knowledge, this gene has not been functionally characterized. In general, few bZIP genes have been functionally characterized in maize [104]. In plants, the bZIP TFs regulate diverse functions, including processes such as plant development and stress response. In our networks analysis, bZIP7 appears as regulator of genes involved in desiccation tolerance, such as glycine-rich protein, hidroxy-proline rich protein, GRAS46, metallothionein 2, glutanione transferase 19, gluthatione transferase 37, anther specific proline rich protein, cupins, SMPs, etc. Therefore, an interesting topic could be to characterize the role of this TF or its homologs.

Unfortunately, in this work we are limited by the lack of data regarding the transcriptomic profile of stage R6, where maturation of the seed ends and the process of desiccation of the caryopsis is almost completed. Further analyses must be carried out during the end of the late embryogenesis phase to uncover the global pattern of disordome in R6 stage.

**Figure 8:**
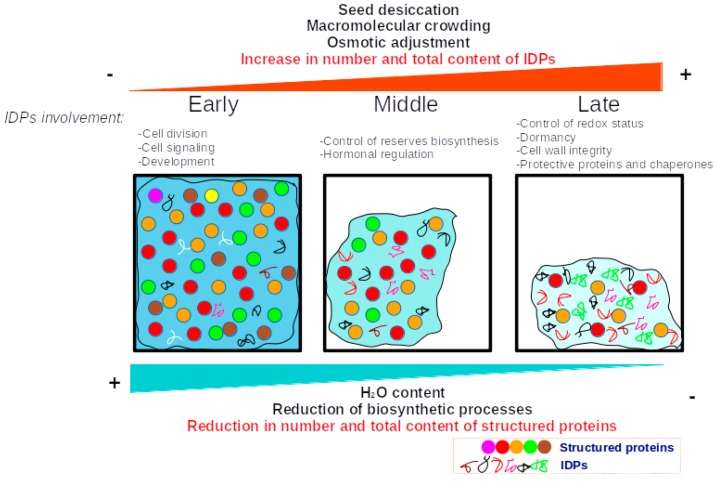
Proposed model of disorder adjustment in the development of orthodox seeds. At the beginning of the development of the seed, the biosynthesis of the reserve compounds and the molecules required to grow and develop is active, the available water is abundant, and the content of structured proteins is high (A). As the development program progresses, there is a significant reduction in water content, cell volume is reduced, and macromolecular crowding increases. This effect causes unwanted interactions between proteins, loss of tertiary structure, and protein aggregation. To reduce this negative effect, cells activate protective mechanisms (such as the biosynthesis of compatible solutes and the induction of chaperones). However, we propose that there is also a sharp reduction of the most labile proteins, without the replacement of other types of structured proteins. In contrast, the content of the IDPs increases and all the genes expressed de novo encodes for IDPs (B). The number of structural proteins continues to decrease, as does their content. It is considered that all this complements the typical mechanisms of tolerance to desiccation. In general, this can be considered to be a strategy to make the proteome more resilient to water-limiting conditions by a mechanism mediated by overall adjustment of the protein disorder.

However, we consider that with the data presented in this work is possible to draw a general model about the alterations that occur during the adjustment process of the disordome as a mechanism of tolerance to desiccation in orthodox seeds. In this proposed model, the content of structured proteins is higher during the most active metabolic stages of seed development, just when the water content is higher. Subsequently, there is a gradual reduction in the number of structured proteins, as well as in their total content. At the same time, the relative content of IDPs increases and with this, the proportion of proteins less prone to losing its function due to loss of structure. This mechanism may facilitate the maintenance of the integrity of the proteome, due to the fact that there would be a lower amount of proteins susceptible to losing its spatial structure, and its molecular function. The general view of such ideas is summarized in Figure 8.

## 5. Conclusions

This study represents the first survey of the dynamics of the expression of transcripts encoding for IDPs in maize seeds. The data obtained in this work help to establish a relationship between global modulation of the overall protein disorder (disordome), and progression of seed development, including the onset of the desiccation tolerance program. In part, such behavior could be explained by the fact that under a desiccation context there is a general reduction in the biosynthetic activities of storage compounds. During this period, we identified several key TFs controlling each stage of seed development and desiccation tolerance acquisition. Interestingly, most of these TFs are IDPs. 

Here, we provide evidence to propose that the onset of desiccation program in seed provoke an actively reduction of proteins that are more prone to loss their function in a water-limiting environment, and an induction of IDPs. The proposed model, although general, could represent an elegant strategy to partially circumvent the negative effects of the compaction effect induced by loss of water, and can be easily extrapolated to other plant and non-plants models in which desiccation tolerance take place. In this sense, the methodological strategy used in this work may be very useful for further studies. 

## Supplementary Materials

The following are available online at https://www.mdpi.com/2073-4425/10/7/502/s1, Table S1: Expression profile of transcripts encoding structured proteins (<25% of protein disorder) during seed development, and their prediction of disorder content, Table S2: Expression profile of transcripts encoding intrinsically disordered proteins (25–50% of protein disorder) during seed development, and their prediction of disorder content, Table S3: Expression profile of transcripts encoding intrinsically disordered proteins (50–75% of protein disorder) during seed development, and their prediction of disorder content, Table S4: Expression profile of transcripts encoding highly disordered proteins (>75% of protein disorder) during seed development, and their prediction of disorder content, Table S5: Complete list of up-regulated transcripts encoding highly intrinsically disordered proteins (>75% of protein disorder) during seed development, Table S6: Complete list of down-regulated transcripts encoding highly intrinsically disordered proteins (>75% of protein disorder) during seed development, Table S7: Complete list of up-regulated key TFs, their target genes and their disorder category that they belong to.
